# Development of a Visible Reverse Transcription-Loop-Mediated Isothermal Amplification Assay for the Detection of Rift Valley Fever Virus

**DOI:** 10.3389/fmicb.2020.590732

**Published:** 2020-11-13

**Authors:** Qiuxue Han, Shengnan Zhang, Dongping Liu, Feihu Yan, Hualei Wang, Pei Huang, Jinhao Bi, Hongli Jin, Na Feng, Zengguo Cao, Yuwei Gao, Hang Chi, Songtao Yang, Yongkun Zhao, Xianzhu Xia

**Affiliations:** ^1^Institute of Laboratory Animal Science, Chinese Academy of Medical Sciences (CAMS) and Comparative Medicine Center, Peking Union Medical College (PUMC), Beijing, China; ^2^Key Laboratory of Jilin Province for Zoonosis Prevention and Control, Institute of Military Veterinary Medicine, Academy of Military Medical Sciences, Changchun, China; ^3^The Nanjing Unicorn Academy of Innovation, Institute Pasteur of Shanghai, Chinese Academy of Sciences, Nanjing, China; ^4^Jiangsu Co-innovation Center for Prevention and Control of Important Animal Infectious Disease and Zoonoses, Yangzhou University, Yangzhou, China; ^5^College of Veterinary Medicine, Jilin University, Changchun, China; ^6^Animal Science and Technology College, Jilin Agricultural University, Changchun, China

**Keywords:** Rift Valley fever virus, reverse transcription-loop-mediated isothermal amplification, nucleic acid visualization, visual detection, inactivated RVFV-BJ01 strain

## Abstract

Rift Valley fever (RVF) is a severe infectious disease, which can through mosquito bites, direct contact and aerosol transmission infect sheep, goats, people, camels, cattle, buffaloes, and so on. In this paper, a conserved region of the S RNA segment of Rift Valley fever virus (RVFV) ZH501 strain was used as target sequence. The RVFV RT-LAMP-VF assay was successfully established combined reverse transcription-loop-mediated isothermal amplification with a vertical flow visualization strip. The detection limit is up to 1.94 × 10^0^ copies/μl of synthesized RVFV-RNA. RNA extracted from cell culture of an inactivated RVFV-BJ01 strain was also used as templates, and the detection limit is 1.83 × 10^3^ copies/μl. In addition, there was no cross-reactivity with other viruses that can cause similar fever symptoms. The RVFV-LAMP-VF assay exhibited very high levels of diagnostic sensitivity, which had 100-fold more sensitive than RVFV real-time RT-PCR assay. Accordingly, the RVFV RT-LAMP-VF assay developed in this study is suitable for the rapid and sensitive diagnosis of RVFV without specialized equipment and can rapidly complete detection within 60 min, and the results are visible by vertical flow visualization strip within 5 min.

## Introduction

Rift Valley fever virus (RVFV) is an arthropod-borne, zoonotic virus (genus *Phlebovirus*, family Phenuivirudae) that is a significant threat to domestic ruminants and humans (Elliott, 1997). RVFV was first discovered in 1930 in Kenya’s Great Rift Valley and spread in Africa (Daubney et al., 1931). The first outbreak outside of Africa was on the Arabian Peninsula in 2000 (Madani et al., 2003; Rolin et al., 2013). As global warming leads to an expanded distribution of insect vectors and increased international trade, the risk of RVFV introduction into countries, where RVFV is not endemic is increasing (Li et al., 2019). Thus, there is urgent demand for the development of safe, rapid, and accurate diagnostic assays.

Techniques for the diagnosis of RVFV include virus isolation, nucleic acid techniques, detection of viral antigen, and specific antibodies. RVFV can be isolated from whole tissues, blood, or serum during the febrile stage of the disease (Crespo Leon et al., 2005). Various serological assays are used to detect antibodies against RVFV (Paweska et al., 2003). Enzyme-linked immunosorbent assays have been extensively validated for the serodiagnosis of RVFV, and an indirect enzyme-linked immunosorbent assay based on the recombinant nucleocapsid protein of RVFV has been recently developed for the detection of specific antibodies in human and animal sera (Paweska et al., 2007, 2008; van Vuren et al., 2007). The virus neutralization test is regarded as the gold standard, and the classical method takes 4–7 days. A RVFV-4seGFP based VNT can be completed in just 48 h (Wichgers Schreur et al., 2017). Moreover, since it requires live virus and can be done only in biocontainment facilities, the virus production requires biocontainment facilities to limit the risk of exposure of laboratory personnel to infection (Paweska et al., 2003). Highly sensitive PCR assays for the detection and quantification of RVFV have been reported, including reverse transcriptase PCR (Garcia et al., 2001; Sall et al., 2002) and real-time RT-PCR (Drosten et al., 2002; Tercero et al., 2019), which require sophisticated equipped laboratories and might be beyond the resources and capabilities of many developing countries. Research on other diagnostic methods has been reported, including optical fiber immunosensor (OFIS), competitive ELISA (Upreti et al., 2018), lateral flow tests (LFT; Cetre-Sossah et al., 2019), fluorescence microsphere immunoassay (FMIA; Ragan et al., 2018), and reverse transcription recombinase polymerase amplification assay (RT-RPA; Euler et al., 2012). The above methods either have low sensitivity, stringent precision requirements and high requirements on operators or present high cost disadvantages. In resource-poor areas, low cost, easy operation, and intuitive reading of results are more important. At present, there is still a lack of a sensitive, intuitive reading method, which is more suitable for on-site testing.

After being infected with RVFV, the initial incubation period is usually 2–6 days, and then enter the fever period, which usually lasts 3–4 days (Mansfield et al., 2015). During the fever period, high levels of viremia occur in humans and animals, and viral RNA can be detected (Ikegami and Makino, 2011). Relevant studies have shown that neutralizing antibodies also begin to appear about the 4th day after the onset of RVF symptoms. IgG and IgM antibodies can be detected at least 6 days after natural infection in humans (Paweska et al., 2005). In a sheep model, IgG and IgM antibodies to RVFV can be detected as early as 4 days after experimental infection (Sobarzo et al., 2007; van Vuren et al., 2007).

For the detection of DNA (Ihira et al., 2004; Okamoto et al., 2004) and RNA (Hong et al., 2004; Mori et al., 2006; Yoda et al., 2007) viruses, differentiation of viral serotypes and subtypes (Parida et al., 2005; Nakagawa and Ito, 2006), and rapid diagnosis of bacterial infections (Iwamoto et al., 2003), the loop-mediated isothermal amplification (LAMP) method has been shown to be highly accurate and sensitive. This procedure requires primers targeting four or six regions of the RNA for amplification. LAMP amplifies target nucleic acid under isothermal conditions, usually between 60 and 65°C. It does not require specialized equipment, offering an inexpensive diagnostic method (Notomi et al., 2000; Le Roux et al., 2009).

This paper reports the development and diagnostic evaluation of an RVFV RT-LAMP with vertical flow visualization strip (RT-LAMP-VF) targeting the S RNA segment of RVFV. We compared the sensitivity of the established RVFV RT-LAMP-VF assay with RVFV real-time RT-PCR.

## Materials and Methods

### Viruses, Synthetic RNA Samples and Mock Infected Blood Sample

The inactivated RVFV-BJ01 strain was gifted by professor Yuhai Bi (Chinese Academy of Sciences, CAS) from the Key Laboratory of Pathogenic Microbiology and Immunology. The target RNA gene was synthesized by Takara Biotechnology (Dalian, China) Co., Ltd., which was selected from a highly conserved region of 270 bp (from 1,336 nt to 1,605 nt) of the RVFV S segment (accession number DQ380149.1), and 70 bp bases were added before and after the gene to prevent RNA degradation.

The mock infected blood sample is made by mixing inactivated RVFV cell culture and fresh volunteer blood at a 1:1 volume ratio. The total RNA was extracted with the TIANamp Virus RNA Kit (TIANGEN, Beijing, China) from 140 μl mixture and eluted with 60 μl of RNase-free water. Above operation were performed in a biosafety level 2+ laboratory.

### Design of Specific Primers for the RVFV RT-LAMP-VF Assay

**Figure 1:**
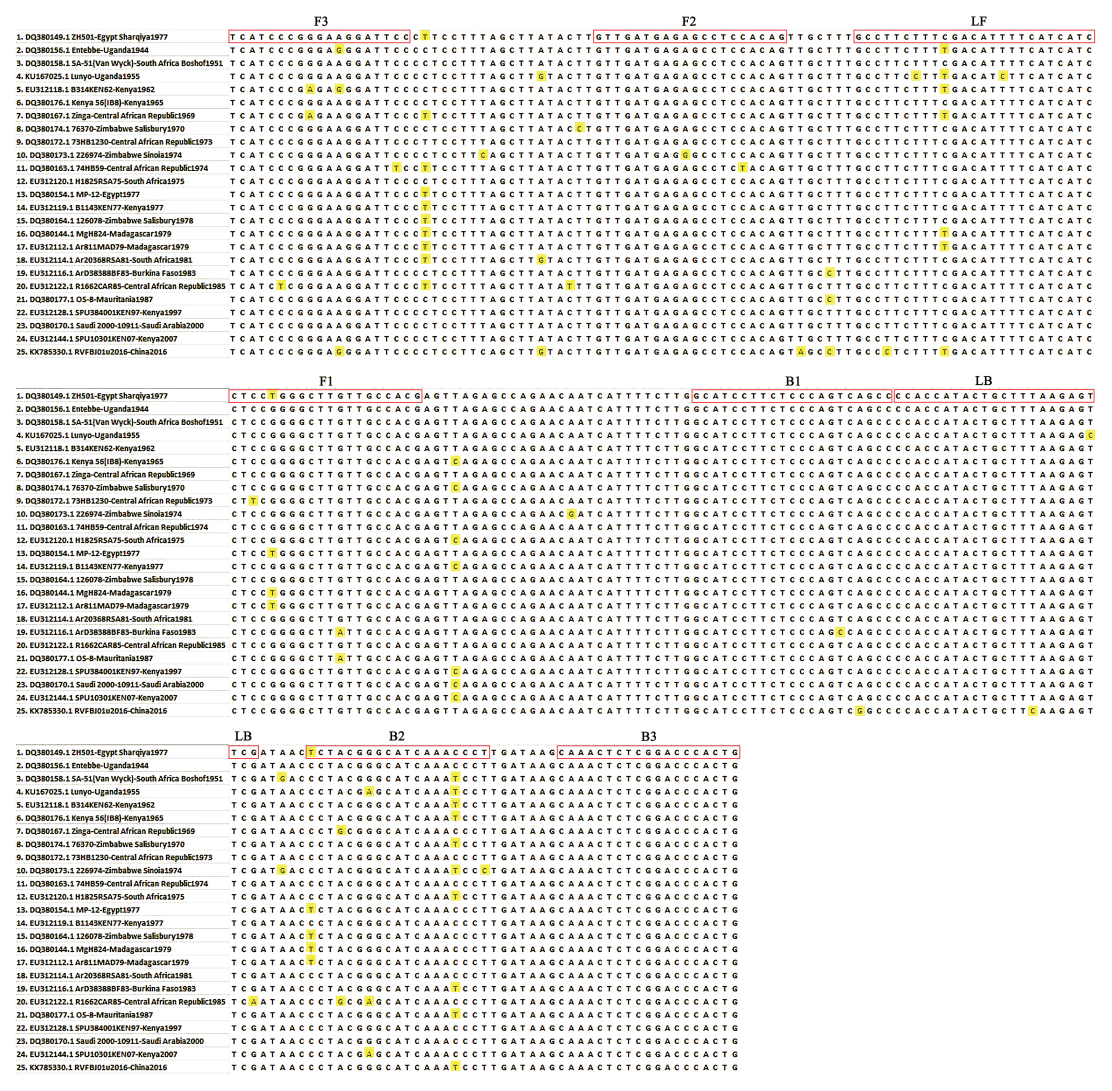
Primer position of the RVFV RT-LAMP-VF assay. The RVFV S segment was retrieved from GenBank and aligned using MEGA 7.0 software.

To establish an RVFV RT-LAMP-VF assay, we designed primers targeting the viral S segment. For primer design, all sequences in the NCBI database (accession numbers DQ380149.1, DQ380156.1, DQ380158.1, KU167025.1, EU312118.1, DQ380176.1, DQ380167.1, DQ380174.1, DQ380172.1, DQ380173.1, DQ380163.1, EU312120.1, DQ380154.1, EU312119.1, DQ380164.1, DQ380144.1, EU312112.1, EU312114.1, EU312116.1, EU312122.1, DQ380177.1, EU312128.1, DQ380170.1, EU312144.1, and KX785330.1) of the full-length RVFV S segment were aligned using MEGA7.0 (Figure 1). Finally, we confirmed the S segment of ZH501 (accession number DQ380149.1) as the target gene and designed RVFV RT-LAMP-VF primers by selecting a highly conserved region of 270 bp from the S segment of ZH501 strain. Six primers (Table 1), including two inner primers (FIP and BIP), two outer primers (F3 and B3), and two loop primers (LF and LB), were designed using LAMP primer software Primer Explorer V5^1^ (2009) and synthesized by Takara Biotechnology (Dalian, China) Co., Ltd. LF and LB primers were labeled by FITC and biotin, respectively, and these two labels can be combined with the corresponding antibodies on the vertical flow visualization strip.

**Table 1:** Primer set for the RT-LAMP-VF assay.

Primer name	Primer position	Sequence (5'-3')
F3	1351–1369	TCATCCCGGGAAGGATTCC
B3	1568–1586	CAGTGGGTCCGAGAGTTTG
FIP (F1c + F2)	1444–1463+	CGTGGCAACAAGCCCAGGAG
	1389–1408	GTTGATGAGAGCCTCCACAG
BIP (B1c + B2)	1492–1512+	GCATCCTTCTCCCAGTCAGCC
	1542–1560	AGGGTTTGATGCCCGTAGA
LF	1416–1440	FITC-GATGATGAAAATGTCGAAAGAAGGC
LB	1513–1536	Biotin-CCACCATACTGCTTTAAGAGTTCG

### RNA Extraction

The RVFV-BJ01 strain was cultured in Vero cells, and the cell culture was collected and inactivated by using beta-propiolactone (BPL; Sigma-Aldrich, St. Louis, Minnesota, United States). The total RNA was extracted with the TIANamp Virus RNA Kit (TIANGEN, Beijing, China) from 140 μl cell fluid and eluted with 60 μl of RNase-free water. All extraction work was performed in a biosafety level 2+ laboratory.

### Pretreatment of Synthesized RNA Transcripts

The RVFV S segment target sequence (from 1,266 to 1,675 nt) (accession number DQ380149.1) was synthesized by Takara Biotechnology (Dalian, China) Co., Ltd. for *in vitro* transcription. First, linearize the plasmid template, use the Takara MiniBEST Agarose Gel DNA Extraction Kit Ver.4.0 to cut the gel to recover the above digestion products, use the Takara *in vitro* Transcription T7 Kit for *in vitro* transcription reaction, and perform the DNase treatment on the RNA transcripts, using Guide-it IVT RNA Clean-Up Kit for refining, DNase treatment again, refining again. After DNase treatment, the RNA transcripts were purified and quantified. The RNA transcripts were measured with a spectrophotometer, and the copy number was calculated using the following formula: copies/μl = 6.02 × 10^23^ × 10^−9^ × concentration/(fragment length × 340). A Nanodrop 2000 ultraviolet visible spectrophotometer was used for quantitative detection of RNA. The concentration of the synthesized RNA transcript was 1,365 ng/μl, which corresponded to 1.94 × 10^11^ copies/μl. The synthesized RNA transcripts were stored at −80°C after purification.

### RVFV RT-LAMP-VF Assay Reaction and Product Detection

Using synthetic RNA of RVFV as a standard template, the isothermal amplification reactions were performed at five different temperatures (59, 61, 63, 65 and 67°C) for 50 min with a 0.4 μM primer (FIP/BIP) concentration. The amplified products were analyzed with the vertical flow visualization strip (Ustar Biotech Co., Ltd., Hangzhou, China) to screen for the optimal temperature. Three replications were performed for each trial. The RNA in the reaction system was replaced with RNase-free water for negative control samples.

Based on the optimum reaction temperature, using the synthetic RNA of RVFV as a standard template, the isothermal amplification reactions were amplified for 20, 30, 40, 50, 60 and 70 min with a 0.4 μM primer (FIP/BIP) concentration, respectively, to obtain the optimal time. The amplified products were analyzed with the vertical flow visualization strip. Three replications were performed for each trial. The RNA in the reaction system was replaced with RNase-free water for negative control samples.

Under the conditions of optimal amplification temperature and time, using synthetic RNA of RVFV as a standard template, the concentration of FIP/BIP primers was set to 0.2, 0.4, and 0.6 μM (LF/LB and F3/B3 concentrations changed proportionally) for constant temperature amplification. The amplified products were analyzed with the vertical flow visualization strip, and the optimal amplification primer concentration was determined. Three replications were performed for each trial. The RNA in the reaction system was replaced with RNase-free water for the negative control samples.

### Quantitative Detection of Inactivated RVFV-BJ01 Strain by Real-Time RT-PCR Assay

Synthetic RVFV-RNA was used as a standard template to establish a real-time RT-PCR method. The real-time RT-PCR primer sequences are shown in Table 2, and the 25 μl reaction mixtures comprised 5 μl of the synthetic RVFV-RNA, 12.5 μl of 2 × Onestep RT-PCR Buffer, 0.5 μl of TakaRa Ex TaqHS as shown in the instructions (Takara Biotechnology, Dalian, China), 0.5 μl of Primer Mix II, 1.0 μl of RVFV-F/R, 2.0 μl of RVFV-Probe, and 2.5 μl RNase-Free water. The optimized amplification conditions were 40 cycles of reverse transcription at 42°C for 5 min; heat denaturation at 95°C for 10 s; 95°C for 90 s, 95°C for 30 s, and 60°C for 45 s. The RNA content of inactivated RVFV cell culture (RVFV-BJ01 strain) was quantified by real-time RT-PCR. The RNA in the reaction system was replaced with RNase-free water as negative control.

**Table 2:** Primer set for the RVFV real-time RT-PCR assay.

Primer name	Primer position	Sequence (5'-3')
RVFV-F	1335–1352	TCGTGATAGAGTCAACTC
RVFV-R	1478–1496	GATGCCAAGAAAATGATTG
RVFV-Probe	1454–1474	FAM-TGGCTCTAACTCGTGGCAACA-TAMRA

### RVFV RT-LAMP-VF Assay Sensitivity and Specificity Evaluation

The synthetic RVFV-RNA was used to assess the sensitivity of the RT-LAMP assay. The synthetic RVFV-RNA with a concentration of 1.94 × 10^11^ copies/μl was diluted 10-fold to 1.94 × 10^−2^ copies/μl. The template was amplified under the optimal reaction conditions to assess the detection limit of the RVFV RT-LAMP assay.

The RNA, which was extracted from inactivated RVFV cell culture was used to assess the sensitivity of the RVFV RT-LAMP-VF assay. The RNA content from inactivated RVFV cell culture with a concentration of 1.83 × 10^7^ copies/μl was diluted 10-fold to 1.83 × 10^1^ copies/μl. The template was amplified under the optimal reaction conditions to assess the detection limit of the RVFV RT-LAMP-VF assay.

The synthesized RVFV-RNA and total RNA extracted from inactivated RVFV cell culture (RVFV-BJ01 strain), inactivated JEV, inactivated H3N2 influenza virus, and recombinant viruses rNDV-EBOV-GP and rSRV9-MGP (expressing MARV G protein) were amplified under the optimal conditions to evaluate the specificity of the RVFV RT-LAMP-VF assay.

Above, the RNase-free water was used to replace the target RNA in the system for negative control samples.

### Analytical Sensitivities of RT-LAMP-VF Assay and Real-Time RT-PCR

The diluted synthesized RVFV-RNA was used as the template. According to the reaction conditions shown above, we compared the analytical sensitivities of the RT-LAMP-VF and real-time RT-PCR with respect to the detection of decreasing numbers of RNA copies under the optimal amplification conditions. Three replications were performed for each trial. The RNA in the reaction system was replaced with RNase-free water for negative control samples.

### Using RVFV Nucleic Acids to Evaluate the RT-LAMP-VF Assay

The RNA extracted from inactivated RVFV cell culture was present at 1.83 × 10^7^ copies/μl as tested by real-time RT-PCR. Inactivated RVFV cell culture was mixed with fresh volunteer blood at a 1:1 volume ratio as a mock infected blood sample, and then the total RNA in the mock infected blood sample was extracted by the QIAamp viral RNA minikit. These samples were amplified under the RVFV RT-LAMP-VF optimal amplification conditions, and three replications were performed for each trial. The RNA extracted from the blood sample was used as the template for the negative control. RNase-free water was used to replace the synthetic RNA for blank control samples.

## Results

### Optimization of Reaction Conditions for the RVFV RT-LAMP-VF Assay

Synthetic RVFV-RNA samples were used as the template to optimize the amplification conditions for the RVFV RT-LAMP-VF assay. To screen the optimal amplification temperature, we amplified the standard templates at different temperatures (57, 59, 61, 63, 65, and 67°C) for 50 min. The results revealed that the optimum amplification temperature was achieved at 63°C. Analysis of the results revealed no significant differences in three replications. The lowest standard template copy value, 0.935 × 10^2^ copies/μl, was detected at 63°C, and so 63°C was considered as the optimum amplification temperature for the RVFV RT-LAMP-VF assay.

To determine the optimum amplification time for the RVFV RT-LAMP-VF assay, synthetic RVFV-RNA samples were amplified for different durations (20, 30, 40, 50, 60 and 70 min) at 63°C, respectively. The results displayed were observed by using the vertical flow visualization strip. The lowest standard template copy value, 1.94 × 10^0^ copies/μl, can be detected at both 60 and 70 min amplification. Therefore, 60 min was selected as the optimal amplification time for the RVFV RT-LAMP-VF assay.

To determine the optimal primer concentration in the amplification, the FIP/BIP concentration was set as 0.2, 0.4, and 0.6 μM (LF/LB and F3/B3 concentrations changed proportionally) to amplify at 63°C for 60 min. As the results indicate, the same results were obtained at 0.4 and 0.6 μM primer concentrations under equivalent conditions. Thus, 0.4 μM for FIP/BIP, 0.2 μM for LF/LB and 0.1 μM for F3/B3 were selected as the optimal primer concentrations for the RVFV RT-LAMP-VF assay.

In summary, the RVFV RT-LAMP-VF assay was established by amplifying for 60 min at 63°C with a FIP/BIP concentration of 0.4 μM.

### Quantitative Analysis of Inactivated RVFV-BJ01 Strain by Real-Time RT-PCR

**Figure 2:**
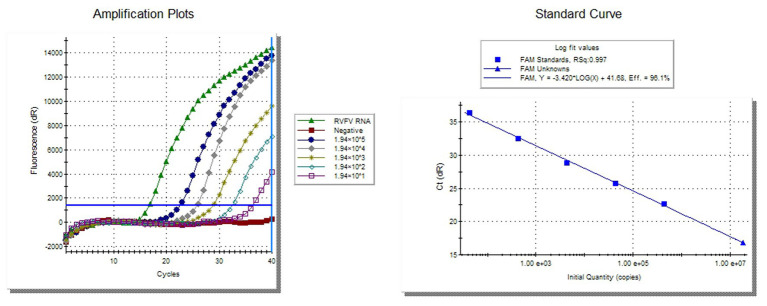
Quantitative detection of inactivated RVFV-BJ01 strain by real-time RT-PCR assay. The left side is the amplification curve and the right side is the standard curve. Dilute the synthesized RNA standard from 1.94 × 10^5^ copies/μl to 1.94 × 10^1^ copies/μl as templates for quantitative detection of RVFV RNA.

The 10-fold diluted synthesized RVFV-RNA was used as the template to establish the standard curve of the RVFV real-time RT-PCR assay. Then, the RNA content of inactivated RVFV-BJ01 was quantified. As the results show in Figure 2, the CT value of inactivated RVFV-BJ01 was 16.84. According to the standard curve *Y* = −3.420*log(x) + 41.68, the RNA content of inactivated RVFV-BJ01 was 1.83 × 10^7^ copies/μl.

### Sensitivity and Specificity of the RT-LAMP-VF Assay

**Figure 3:**
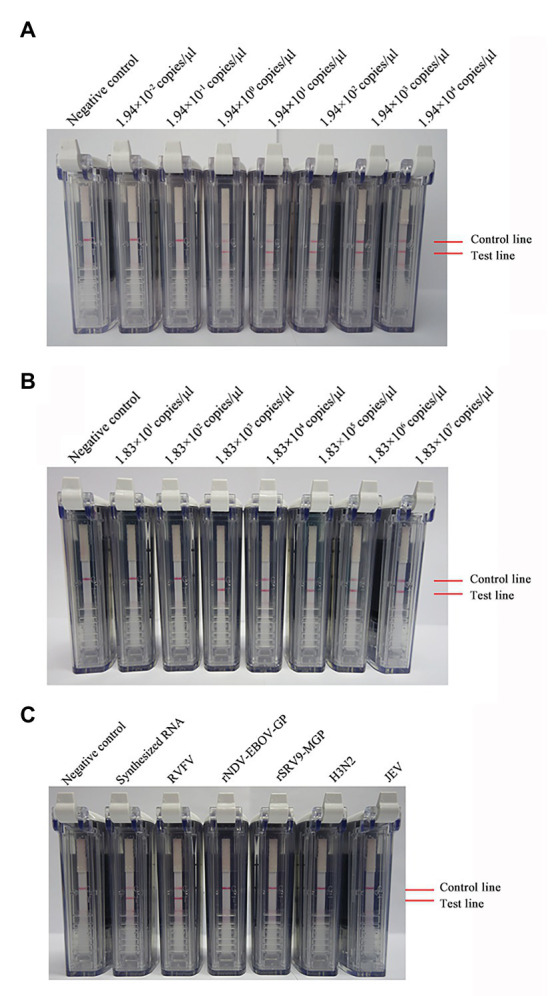
Detection of the sensitivity and specificity of the RVFV RT-LAMP-VF assay. Sensitivity evaluation of the RVFV RT-LAMP-VF assay by using a series of synthesized RVFV-RNA **(A)** and RNA extracted from inactivated RVFV-BJ01 strain **(B)**. Specificity evaluation of the RVFV RT-LAMP-VF assay **(C)**.

Ten-fold dilutions of synthesized RNA (ranging from 1.94 × 10^4^ to 1.94 × 10^−2^ copies/μl) were used to evaluate the sensitivity of the RVFV RT-LAMP-VF assay. As Figure 3A shows, the assay limit of detection for synthesized RNA was 1.94 × 10^0^ copies/μl within 60 min.

Ten-fold dilutions of the RNA extracted from inactivated RVFV cell culture (ranging from 1.83 × 10^7^ to 1.83 × 10^1^ copies/μl) were used to evaluate the sensitivity of the RVFV RT-LAMP-VF assay. As Figure 3B shows, the assay limit of detection for inactivated RVFV-BJ01 RNA was 1.83 × 10^3^ copies/μl within 60 min.

Subsequently, the RNAs from viruses with similar clinical symptoms to RVFV and other laboratory stock viruses were used to evaluate the specificity of the RVFV RT-LAMP-VF assay. As seen from the results shown in Figure 3C, the result is positive only when the synthetic RVFV-RNA and inactivated RVFV-BJ01 RNA were used as templates. Consequently, the RVFV RT-LAMP-VF assay had good specificity.

### Compared Sensitivity of RVFV RT-LAMP-VF Assay and Real-Time RT-PCR

**Figure 4:**
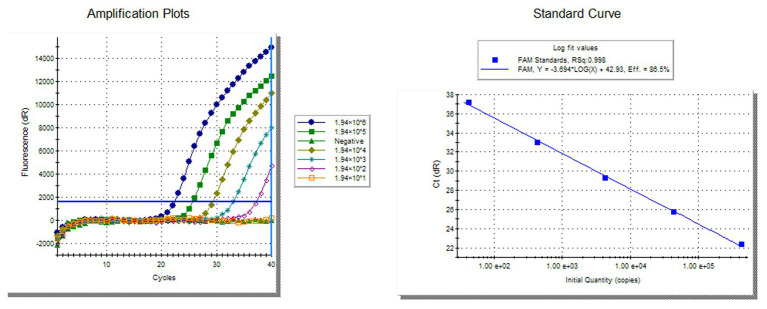
Compared sensitivity of RVFV RT-LAMP-VF assay and RVFV real-time RT-PCR. Ten-fold diluted the synthesized RNA standard as templates for RVFV real-time RT-PCR. The left side is the amplification curve and the right side is the standard curve.

To compare the sensitivity of the RVFV RT-LAMP-VF assay and real-time RT-PCR, the synthesized RNA standard was used as the template, which was diluted 10 times to 1.94 × 10^−2^ copies/μl. The limit of detection by RVFV RT-LAMP-VF was 1.94 × 10^0^ copies/μl (Figure 3A), and the limit of detection by real-time RT-PCR was 1.94 × 10^2^ copies/μl (Figure 4). These data demonstrated that the RVFV RT-LAMP-VF assay was 100-fold more sensitive than the real-time RT-PCR assay.

### Diagnostic Evaluation of the RVFV RT-LAMP-VF Assay

**Figure 5:**
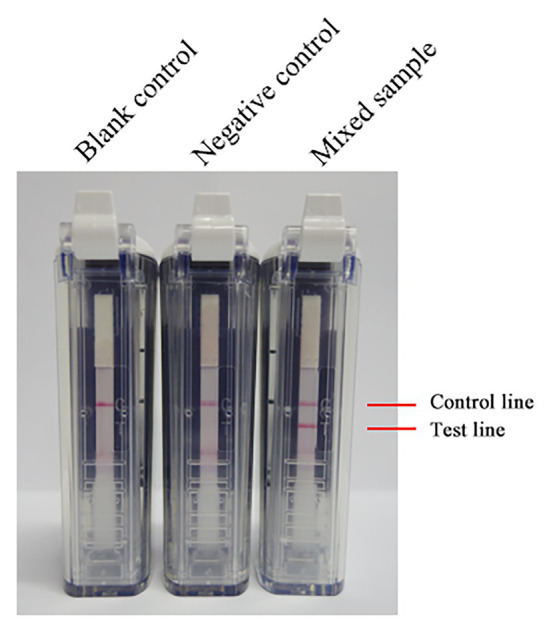
The RNA of inactivated RVFV cell culture (RVFV-BJ01 strain) was used to evaluate the RVFV RT-LAMP-VF assay. Inactivated RVFV cell culture was mixed with fresh volunteer blood at a 1:1 volume ratio as a mock infected blood sample to simulate clinical samples.

After mixing the inactivated RVFV cell culture with fresh blood of a volunteer, total RNA in the mixture was extracted by using a viral RNA extraction kit. The total RNA was amplified at 63°C with 0.4 μM primer concentration for 60 min. As Figure 5 shows, RNA in the mixed sample can be detected. It is preliminarily indicated that the RVFV RT-LAMP-VF assay can be applied to clinical sample detection.

## Discussion

RVFV is an important zoonotic disease and poses a potential bioterrorism threat. Many kinds of mosquito vectors could transmit RVFV, and the effects of global climate change could facilitate the spread of arthropod-borne viruses into nonendemic countries. The potential for further spread of RVFV outside its traditional geographic boundaries has resulted in increased international demand for validated molecular tools for the rapid and accurate diagnosis of RVFV.

LAMP is a novel nucleic acid detection method invented in 2000 (Notomi et al., 2000). This method relies on strand displacement DNA polymerase with high strand displacement activity and a set of two specially designed inner and two outer primers. First, the inner primers initiates LAMP then the target sequence is amplified to form a new single-strand of DNA. Under the action of strand displacement DNA polymerase, the outer primer releases a single-stranded of DNA. The released single strand and the special structure of the inner primer will fold to form cauliflower-like structures. After a series of amplification, a product with multiple ring structures will be formed (Cao et al., 2016; Huang et al., 2018). For the RVFV RT-LAMP-VF assay, in order to be combined with the vertical flow visualization strip, FITC and biotin were labeled at the 5' ends of the loop primers LF and LB, respectively. In addition, given that this experiment uses RNA as templates, so AMV Reverse Transcriptase (Promega) was added to the reaction system. Because LAMP recognizes the target by six distinct sequences, it is expected to amplify the target sequence with high specificity.

The vertical flow visualization test was used to read the results, using the principle of double antibody sandwich. It consisted of test strips and diluent. The quality control line and the detection line of the strip were marked with anti-streptavidin antibody and anti-FITC antibody, respectively. At the same time, gold particles covered with streptavidin were incubated on the binding pad of the strip. The primers LF and LB were covered with FITC and biotin, respectively. After the amplified product was added to the device, the gold particles would be combined with the biotin on the product through streptavidin under the action of the diluent and migrated upward to the detection line under the siphon action of the strip. The FITC on the product would bind to the anti-FITC antibody on the detection line, and a large number of gold particles would aggregate to form a visible red detection line.

RVFV RT-LAMP has been established, but it still has some technical shortcomings, such as false positive problems and required special equipment (turbidity meter). When reading the results, the agarose gel electrophoresis method is mostly used, presenting the problem of false positives (Peyrefitte et al., 2008; Le Roux et al., 2009). In addition, there are subjective differences when reading the results of precipitation and staining methods. In this study, the RVFV RT-LAMP-VF technique for rapid and accurate detection of RVFV RNA was investigated. In addition to the high levels of analytical and diagnostic accuracy and speed of detection, another important practical advantage of the RVFV RT-LAMP-VF assay is that it uses simple and relatively inexpensive equipment, which renders it promising for use in resource-poor settings. In addition, only basic molecular and technical skills are required for the assay procedure, and the results could be identified by the naked eye. The primer design for the RVFV RT-LAMP-VF assay is more complex than for the conventional PCR-based assays and, in this study, we designed the RVFV RT-LAMP-VF primers to target the S segment of RVFV, which encodes N protein and is highly conserved among RVFV strains. In Figure 1, we can see that there are unmatched nucleotides (highlighted part) between different RVFV strains, which are also present in the primer sequence. The influence of these differences on the sensitivity of the assay may exist, and the specific degree of influence remains to be demonstrated. However, in order to avoid the difference of nucleotides from affecting the detection sensitivity as much as possible, when screening conservative sequences in the early stage, we selected strains from different species, different regions, and different ages for comparison.

The synthesized RVFV-RNA was used as the template to determine the optimal amplifications, including temperature and time and the concentration of primers. Among the results demonstrated in this paper, 63°C was considered as the optimum amplification temperature and 60 min was selected as the optimal amplification time; 0.4 μM FIP/BIP, 0.2 μM LF/LB, and 0.1 μM F3/B3 were selected as the optimal primer concentrations.

In this study, we compared the sensitivity of the RVFV RT-LAMP-RV assay and RVFV real-time RT-PCR assay. The results of the study showed that the RVFV RT-LAMP-VF assay (1.94 × 10^0^ copies/μl) was 100-fold more sensitive than the RVFV real-time RT-PCR assay (1.94 × 10^2^ copies/μl). RVFV RT-LAMP-VF was subjected to specific evaluation by detecting viruses with similar clinical symptoms (like JEV, H3N2 influenza virus, EBOV, and MARV). The results indicated no cross-reaction with inactivated JEV, inactivated H3N2 influenza virus, and recombinant virus rNDV-EBOV-GP and rSRV9-MGP.

Because the collected clinical RVFV samples were mostly blood, we mixed volunteer blood with inactivated RVFV cell culture at a 1:1 volume ratio to simulate a clinical sample, which was subjected to the RVFV RT-LAMP-VF assay. The results indicated that the sensitivity and specificity of RVFV RT-LAMP-VF were not interfered with by other components in the blood sample.

RT-LAMP is an inexpensive and sensitive detection method for viral RNA and is suitable for field surveys, where specialized equipment is often unavailable. RT-LAMP assays for Ebola virus (Kurosaki et al., 2007), Zika virus (Kurosaki et al., 2017), and Chikungunya virus (Lopez-Jimena et al., 2018), for which diagnostics are necessary in remote areas, have been developed. Under the ravages of SARS-CoV-2, LAMP-based detection methods have also been developed (Yan et al., 2020). Large-scale epidemiological studies are important to predict virus epidemics and transmission routes, thereby facilitating the development of countermeasures against viruses.

One specific conclusion is that the established RVFV RT-LAMP-VF assay has a detection limit of 1.94 × 10^0^ copies/μl RNA transcripts and 1.83 × 10^3^ copies/μl viral RNA. This method offers the advantages of high sensitivity, strong specificity, visual reading, and easy operation. Hence, the RVFV RT-LAMP-VF assay has the potential for rapid detection on point-care-testing. Since the entire amplification process is carried out under constant temperature conditions, only a simple water bath or metal bath is needed. In addition, after the reaction solution is added to the PCR tube, there is no need to open the cap to read the result, which avoids false positives caused by aerosol contamination, and the vertical flow visualization strip has the advantages of simple operation and easy portability. This makes this method highly practical, especially for insufficient equipment areas. Based on the above experimental results, from the perspective of epidemiological investigation, epidemic prevention, and control and technology reserve of Rift Valley fever, the establishment of the RVFV RT-LAMP-VF assay is of great significance. With the development of technology, a digital diagnostic method with rapid execution, high accuracy, sensitivity and specificity, low cost, and suitability for on-site diagnosis will become a trend.

## Data Availability Statement

The original contributions presented in the study are included in the article/supplementary material, further inquiries can be directed to the corresponding authors.

## Ethics Statement

The studies involving human participants were reviewed and approved by Use Committee of the Chinese People’s Liberation Army. The patients/participants provided their written informed consent to participate in this study.

## Author Contributions

YZ, YG, and XX designed the experiments. QH, SZ, PH, FY, JB, ZC, and HC performed the experimentation. QH, DL, HW, NF, and HJ analyzed the data. QH and SZ wrote the manuscript. All authors contributed to the article and approved the submitted version.

## Conflict of Interest

The authors declare that the research was conducted in the absence of any commercial or financial relationships that could be construed as a potential conflict of interest.

## Footnotes

^1^http://primerexplorer.jp/lampv5e/index.html
